# Supplementary material for: Protein-crystal detection with a compact multimodal multiphoton microscope
Source: Commun Biol. 2020 Oct 13;3:569. doi: 10.1038/s42003-020-01275-8 (PMC7553921; doi:10.1038/s42003-020-01275-8)
Supplement: Supplementary file 1 — Supplementary Information [file 42003_2020_1275_MOESM1_ESM.pdf]

# Supplementary Information

## Protein-crystal detection with a compact multimodal multiphoton microscope

Qing-di Cheng, Hsiang-Yu Chung, Robin Schubert, Shih-Hsuan Chia, Sven Falke, Celestin Nzanzu Mudogo, Franz X. Kärtner, Guoqing Chang and Christian Betzel

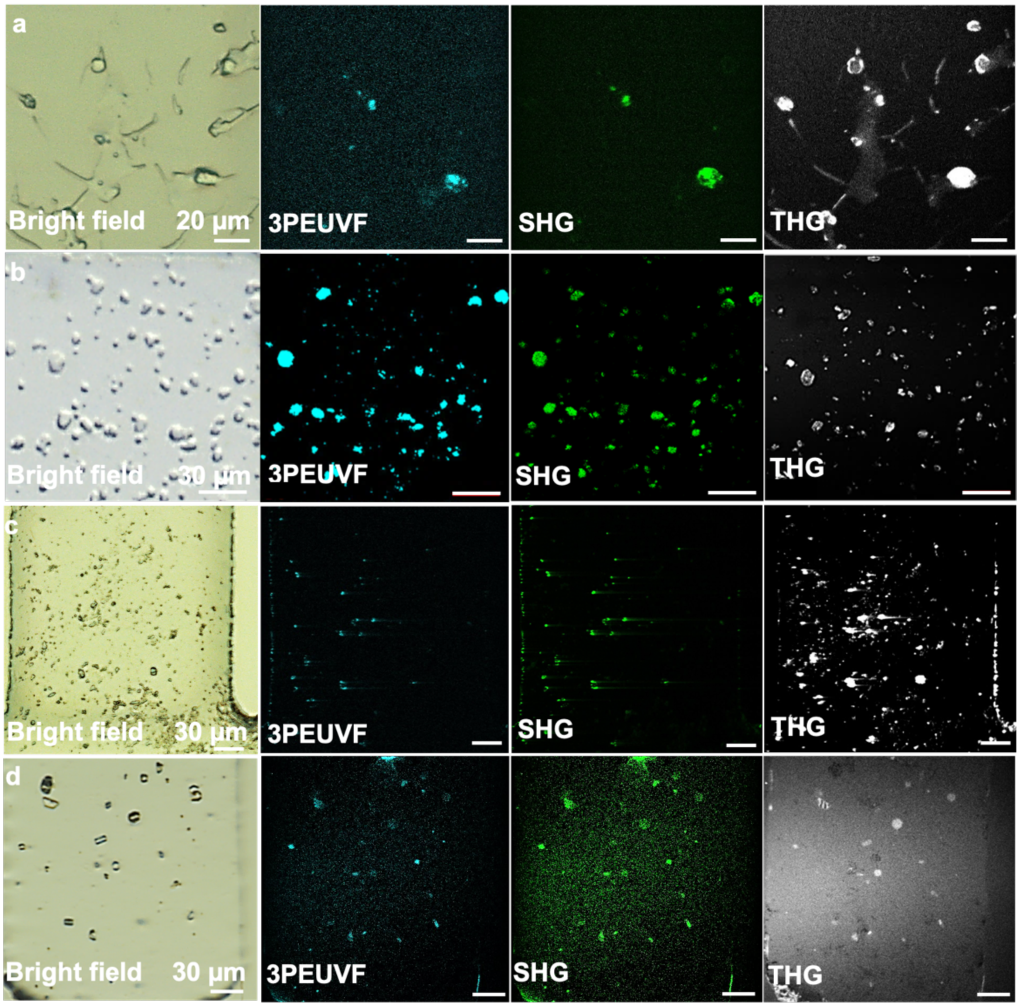


**Supplementary Figure 1 Images recorded for different protein crystals applying the MPM system.** The method of detection and imaging is indicated in the lower left of each image. (a)-(d) MPM imaging of glucose isomerase, bovine serum albumin, insulin and lactamase.

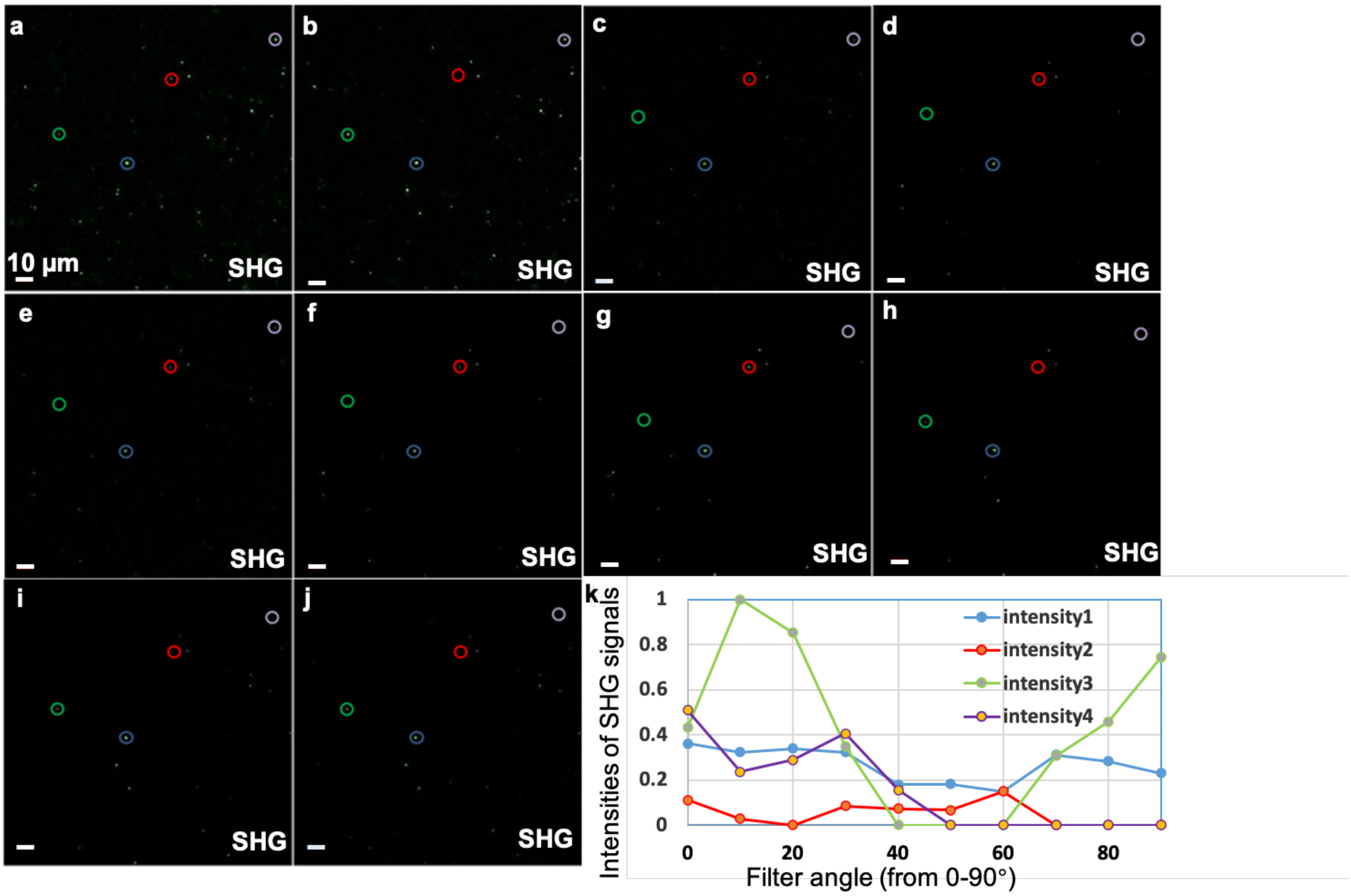


**Supplementary Figure 2 Polarization-dependent SHG signal intensities of images taken from thaumatin crystals.** (a)-(j) Angular dependence of recorded SHG intensities applying a suspension of thaumatin crystals and changing the polarization plane in steps of 10° from 0-90° by rotating a half-wave plate (HWP) in the MPM system. The scale bar corresponds to 10 µm.

(k) Polarization-dependent line chart of the recorded SHG signal intensities.

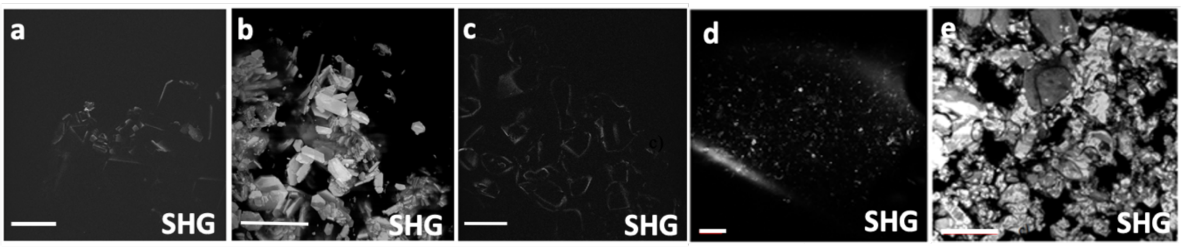


**Supplementary Figure 3 SHG images of salt crystals recorded by the MPM instrument.** (a) NaCl. (b) $Li_2SO_4 \cdot H_2O$. (c) $Na_2SO_4$. (d) KCl. (e) $KH_2PO_4$ (the scale bar corresponds to 50 μm).

## Supplementary Table1

### Crystallization conditions and space groups of used proteins

| Protein | Buffer solution | Protein concentration (mg/ml) | Precipitant | Space group |
| --- | --- | --- | --- | --- |
| Lysozyme (chicken egg white) | 100 mM sodium acetate, pH 4.5 | 100 | 4.5 M sodium chloride, 5% PEG 8000, 0.1 M sodium acetate, pH 4.5 | P $4_3 2_1 2$ |
| Proteinase K (Tritirachium album) | 20 mM MES-NaOH, pH 6.5 | 40 | 1 M sodium nitrate, 0.1 M calcium chlorrie, 0.1 M MES-NaOH, pH 6.5 | P $4_3 2_1 2$ |
| Thaumatin (Thaumatococcus daniellii) | 50 mM Bis-tris, pH 6.5 | 30 | 2 M sodium tartrate, 50 mM Bis-tris, pH 6.5 | P $4_1 2_1 2$ |
| Thermolysin (Geobacillus stearothermophilus) | 45% DMSO, 2.5M caesium chloride, 50mM Tris, pH 7.0 | 45 | $H_2O$ | P $6_1$ 2 2 |
| Glucose Isomerase (Streptomyces murinus) | 10 mM HEPES, pH 7.0 | 30 | 2 M ammonium sulfate, 50 mM HEPES, pH 7.0 | I 2 2 2 |
| Bovine serum albumin (bovine serum) | 0.1 M Tris HCl, pH 6.5 | 20 | 0.2 M Ca acetate, 20% PEG3350, 0.1 M Tris HCl, pH 6.5 | C 2 |
| Insulin (bovine pancreas) | 50 mM sodium phosphate, 10 mM EDTA, pH 10.0 | 20 | 0.5 M sodium phosphate, pH 10.0 | I $2_1$ 3 |
| Lactamase (purified in house) | 20 mM MES, 50mM NaCl, pH 4.5 | 7 | 25% PEG 8000, 0.2 M $Li_2SO_4$, 0.1 M Na Acetat, pH 4.5 | P $3_2 21$ |

# Supplementary Table 2

## Details for salt crystals used for the experiments

| Salt crystals | Space group | Centrosymmetric | MPM recorded SHG signal yes/no | MPM recorded SHG signal activity | Corresponding references about SHG signals |
|---|---|---|---|---|---|
| NaCl | F m-3m | Yes | Yes | ** | No SHG [1] |
| Ca $(CH_3COO)_2 \cdot H_2O$ | P-1 | Yes | No | | No report |
| $NaKC_4H_4O_6 \cdot 4H_2O$ (Sodium Potassium Tartrate Tetrahydrate) | P $2_12_12_1$ | No | Yes | * | Yes [1] |
| $Li_2SO_4 \cdot H_2O$ | P $2_1$ | No | Yes | *** | Yes [1,2] |
| $CaCl_2$ | P n n m | Yes | No | | No [1] |
| KCl | F m-3m | Yes | Yes | ** | No [1] |
| $Na_3C_6H_5O_7 \cdot 2H_2O$ (Sodium citrate dihydrate) | C2/c | Yes | No | | No [1] |
| $KH_2PO_4$ | I-42d | No | Yes | *** | Yes [1,3] |
| $NH_4Cl$ | P m-3m | Yes | Yes | * | No [1] |
| $MgCl_2$ | R -3m | Yes | Yes | * | No report |
| $NaH_2PO_4$ | P $2_1$/c | Yes | No | | No [1] |
| $NaNO_3$ | R -3c | Yes | No | | No report |
| $(NH_4)_2SO_4$ | P n m a | Yes | No | | No [1] |
| $NH_4H_2PO_4$ | I-42d | No | Yes | * | Yes [1] |
| $Na_2SO_4$ | F ddd | Yes | Yes | ** | No [1] |

In the Table 2, * indicates weak, ** strong, *** very strong signals

# Supplementary Table 3

## MPM modalities applied in this paper for protein crystal detection.

| Modality | Contrast agent | Excitation wavelength ($\lambda_{ex}$) | Emission wavelength ($\lambda_{em}$) |
|---|---|---|---|
| SHG | Optical non-centrosymmetry | Not fixed | 1/2 of $\lambda_{ex}$ |
| THG | Optical inhomogeneity | Not fixed | 1/3 of $\lambda_{ex}$ |
| 2PEUVF | Chromophore in AAAs | ~532 nm | ~350 nm |
| 3PEUVF | Chromophore in AAAs | ~800 nm | ~350 nm |

SHG: second-harmonic generation, THG: third-harmonic generation, 2PEUVF/3PEUVF: two-/three-photon excited ultraviolet fluorescence, AAA: aromatic amino acid

# Supplementary Table 4

### Comparison of parameters between the SONICC and the in-house built MPM system

| | SONICC System | In-house built MPM System |
|---|---|---|
| Laser repetition rate | 80 MHz | 31 MHz |
| Objective NA | 0.51 | 1.05 |
| Pulse duration | 200 fs | 100-200 fs |
| Maximum FOV | 0.65 x 0.65 $mm^2$ | 0.8 x 0.8 $mm^2$ |

The in-house developed MPM system has the following advantages over sensitivity compared to the commercial SONICC system:
1. The NA of the objective lens is higher, which results in a smaller focus with higher laser intensity.
2. The pulse duration can be tuned to be shorter.
3. At the same excitation power, the pulse energy and peak power are higher due to the lower laser repetition rate.

# Supplementary Table 5

### Specification for sample containers and imaging conditions

| Containers | Volume of mixing solution | Inflow (yes/no) | exposure time (s) |
|---|---|---|---|
| LCP plates | 1 µl (in each well) | no | 3-5 |
| PMDS microfluidic chips | 100 µl (in each plate) | yes | 3-5 |
| Swissci 96-well 2-drop crystallization plates | 1 µl (in each well) | no | 3-5 |
| Cover glass | 1 µl | no | 4-5 |
| Capillary | 100 µl | yes | 3-5 |

# Supplementary References


1. Closser, R. G., Gualtieri, E. J., Newman, J. A. & Simpson, G. J. Characterization of salt interferences in second-harmonic generation detection of protein crystals. *J. Appl. Crystallog*. **46**, 1903-1906 (2013).
2. Bayarjargal, L. Nonlinear optical properties of lithium sulfate monohydrate. *Cryst. Res.Technol*. **43**, 1138-1142 (2008).
3. Imasaka, T., Tashiro, K. & Ishibashi, N. Analytical instrumentation based on optical harmonic generation. *Anal. Sci*. **6**, 301-302 (1990).
