# Supplementary material for: Protein-crystal detection with a compact multimodal multiphoton microscope
Source: Commun Biol. 2020 Oct 13;3:569. doi: 10.1038/s42003-020-01275-8 (PMC7553921; doi:10.1038/s42003-020-01275-8)
Supplement: Supplementary file 3 — Supplementary Movie 1 [file 42003_2020_1275_MOESM3_ESM.pptx]

## Slide 1
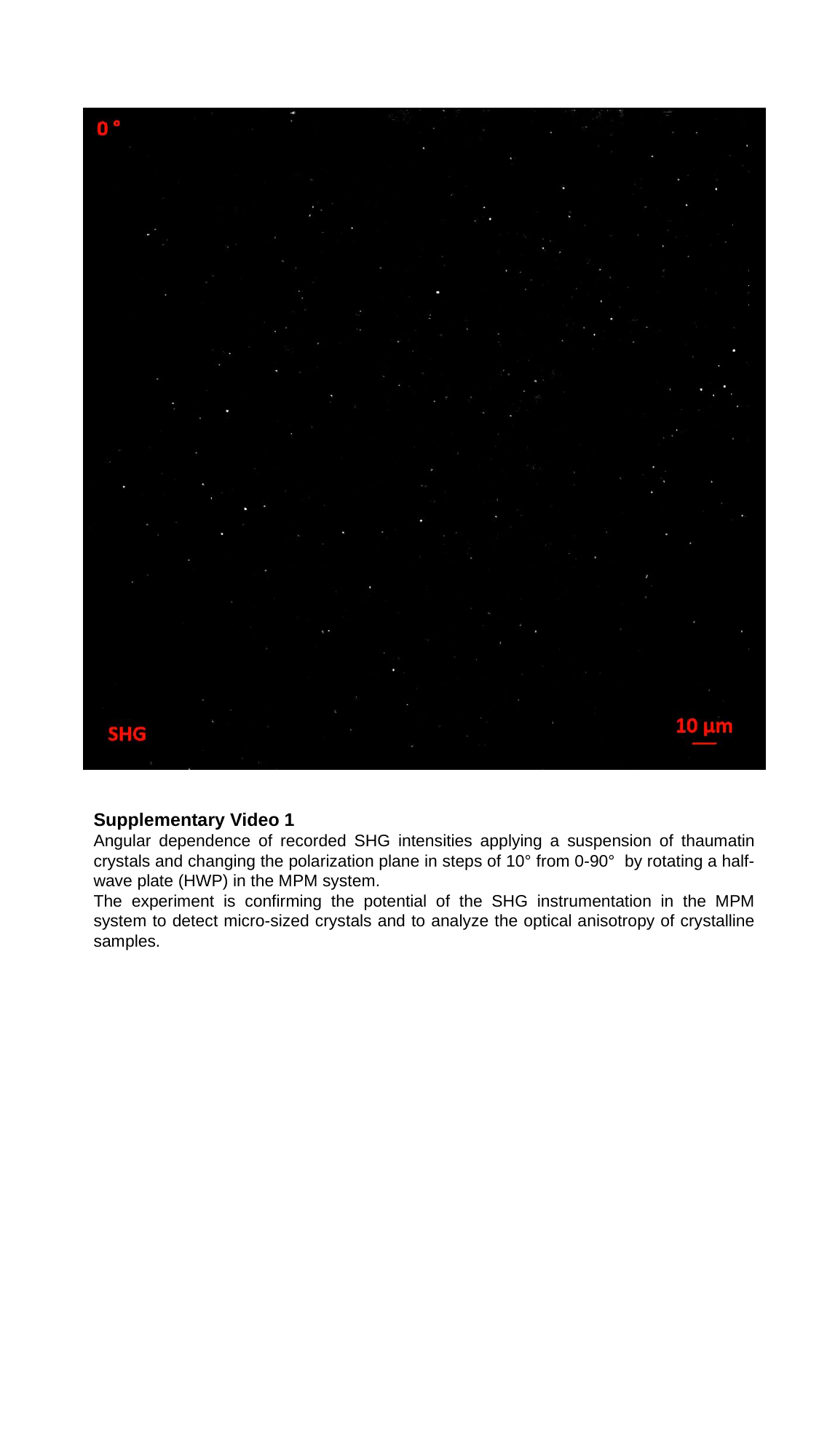

Supplementary Video 1
Angular dependence of recorded SHG intensities applying a suspension of thaumatin crystals and changing the polarization plane in steps of 10° from 0-90° by rotating a half-wave plate (HWP) in the MPM system.
The experiment is confirming the potential of the SHG instrumentation in the MPM system to detect micro-sized crystals and to analyze the optical anisotropy of crystalline samples.
