# Supplementary material for: Protein-crystal detection with a compact multimodal multiphoton microscope
Source: Commun Biol. 2020 Oct 13;3:569. doi: 10.1038/s42003-020-01275-8 (PMC7553921; doi:10.1038/s42003-020-01275-8)
Supplement: Supplementary file 4 — Reporting Summary [file 42003_2020_1275_MOESM4_ESM.pdf]

nature research

Corresponding author(s): C.Q., Ch.B., F.X.K.

Last updated by author(s): Aug 12, 2020

# Reporting Summary

Nature Research wishes to improve the reproducibility of the work that we publish. This form provides structure for consistency and transparency in reporting. For further information on Nature Research policies, see our Editorial Policies and the Editorial Policy Checklist.

## Statistics

For all statistical analyses, confirm that the following items are present in the figure legend, table legend, main text, or Methods section.

| n/a | Confirmed | |
|---|---|---|
| ☐ | ☒ | The exact sample size (*n*) for each experimental group/condition, given as a discrete number and unit of measurement |
| ☐ | ☒ | A statement on whether measurements were taken from distinct samples or whether the same sample was measured repeatedly |
| ☒ | ☐ | The statistical test(s) used AND whether they are one- or two-sided<br>*Only common tests should be described solely by name; describe more complex techniques in the Methods section.* |
| ☒ | ☐ | A description of all covariates tested |
| ☒ | ☐ | A description of any assumptions or corrections, such as tests of normality and adjustment for multiple comparisons |
| ☒ | ☐ | A full description of the statistical parameters including central tendency (e.g. means) or other basic estimates (e.g. regression coefficient) AND variation (e.g. standard deviation) or associated estimates of uncertainty (e.g. confidence intervals) |
| ☒ | ☐ | For null hypothesis testing, the test statistic (e.g. *F*, *t*, *r*) with confidence intervals, effect sizes, degrees of freedom and *P* value noted<br>*Give P values as exact values whenever suitable.* |
| ☒ | ☐ | For Bayesian analysis, information on the choice of priors and Markov chain Monte Carlo settings |
| ☒ | ☐ | For hierarchical and complex designs, identification of the appropriate level for tests and full reporting of outcomes |
| ☒ | ☐ | Estimates of effect sizes (e.g. Cohen's *d*, Pearson's *r*), indicating how they were calculated |

*Our web collection on statistics for biologists contains articles on many of the points above.*

## Software and code

Policy information about availability of computer code

| | |
|---|---|
| Data collection | To operate the MPM microscope the Thorlabs software suite, ThorImageLS, an open-source image acquisition program that controls Thorlab's microscope set ups was used. This software is available via https://www.thorlabs.com/newgrouppage9.cfm?objectgroup_id=9072. |
| Data analysis | The software ImageJ for pseudo-color can be found at https://imagej.net/Welcome. |

For manuscripts utilizing custom algorithms or software that are central to the research but not yet described in published literature, software must be made available to editors and reviewers. We strongly encourage code deposition in a community repository (e.g. GitHub). See the Nature Research guidelines for submitting code & software for further information.

## Data

Policy information about availability of data

All manuscripts must include a data availability statement. This statement should provide the following information, where applicable:

- Accession codes, unique identifiers, or web links for publicly available datasets
- A list of figures that have associated raw data
- A description of any restrictions on data availability

Images and data generated during the study are available from the corresponding authors upon request.

# Field-specific reporting

Please select the one below that is the best fit for your research. If you are not sure, read the appropriate sections before making your selection.

☒ Life sciences ☐ Behavioural & social sciences ☐ Ecological, evolutionary & environmental sciences

For a reference copy of the document with all sections, see nature.com/documents/nr-reporting-summary-flat.pdf

# Life sciences study design

All studies must disclose on these points even when the disclosure is negative.

| | |
|---|---|
| Sample size | We utilized micro- and nanocrystals from approx. 500 nm to 10 micrometer. |
| Data exclusions | We have not excluded data or images. |
| Replication | During construction and commissioning of the MPM microscope we applied in vitro and in vivo grown crystals, as described in the manuscript to analyse and verify the performance of the intrument. And all measurements were reproducible. |
| Randomization | To test and verify the performance and the SHG and THG detection capability of the MPM microscope we applied protein and salt crystals with distinct space groups and internal symmetries, as well as having different dimensions and volumes. |
| Blinding | We were not blinded to group allocation during data collection, since our tagret samples are micro- or nanocrystals. These crystals with small dimensions were selected to be detected and analysed |

# Reporting for specific materials, systems and methods

We require information from authors about some types of materials, experimental systems and methods used in many studies. Here, indicate whether each material, system or method listed is relevant to your study. If you are not sure if a list item applies to your research, read the appropriate section before selecting a response.

## Materials & experimental systems

| n/a | Involved in the study | |
|---|---|---|
| ☒ | ☐ | Antibodies |
| ☐ | ☒ | Eukaryotic cell lines |
| ☒ | ☐ | Palaeontology and archaeology |
| ☒ | ☐ | Animals and other organisms |
| ☒ | ☐ | Human research participants |
| ☒ | ☐ | Clinical data |
| ☒ | ☐ | Dual use research of concern |

## Methods

| n/a | Involved in the study | |
|---|---|---|
| ☒ | ☐ | ChIP-seq |
| ☒ | ☐ | Flow cytometry |
| ☒ | ☐ | MRI-based neuroimaging |

## Eukaryotic cell lines

Policy information about cell lines

| | |
|---|---|
| Cell line source(s) | Sf9 cells, Thermo fisher scientific; HEK 293 cells, Thermo fisher scientific. |
| Authentication | None of the cell lines were authenticated. |
| Mycoplasma contamination | HEK293 cells have been checked by mycoplasma contamination, and the HEK 293 cells were negative |
| Commonly misidentified lines (See ICLAC register) | No mis-idenfitied cell lines were used in this work. |
